# Impact of eFOV CT distortion on surface‐guided radiotherapy registration: Phantom‐based analysis

**DOI:** 10.1002/acm2.70312

**Published:** 2025-10-26

**Authors:** Wooseok Kim, Hyunsoo Jang, Eng Chan Kim, Jonggeun Baek, Byungyong Kim

**Affiliations:** ^1^ Department of Radiation Oncology Pohang St. Mary's Hospital Pohang‐si Gyeongsangbuk‐do Republic of Korea; ^2^ Department of Physics Yeungnam University Gyeongsan‐si Gyeongsangbuk‐do Republic of Korea; ^3^ Department of Radiation Oncology Dongguk University Gyeongju Hospital Gyeongju‐si Gyeongsangbuk‐do Republic of Korea; ^4^ Department of Radiation Oncology Keimyung University Dongsan Hospital Dalseo‐gu Daegu Republic of Korea

**Keywords:** extended field‐of‐view, image registration, reference surface, surface‐guided radiation therapy

## Abstract

**Purpose:**

This study evaluated the impact of extended field‐of‐view (eFOV) CT distortion on the alignment accuracy of surface‐guided radiation therapy (SGRT) using CT‐based reference surfaces, and examined the influence of beam center location.

**Methods:**

An anthropomorphic phantom, segmented into chest, abdomen, and pelvis, was scanned at the center position (sFOV) and at four off‐center positions. The center corresponds to the CT bore isocenter, while the off‐center positions were obtained by placing the phantom boundary 315 mm or 390 mm to the left or right of the CT isocenter to simulate eFOV conditions. Geometric distortion was assessed by measuring external body contour volume. Treatment plans were generated with beam centers at the isocenter and at off‐center positions of 8 cm left and right. SGRT registration accuracy was evaluated from SGRT alignment error and cone‐beam CT (CBCT) alignment error differences using axis‐specific mean absolute errors (MAEs) and overall translational and rotational root mean square errors (RMSEs).

**Results:**

Distortion was most pronounced in the chest when the phantom boundary was 390 mm right of the isocenter, with a 12.55% volume reduction. In contrast, abdomen and pelvis volume changes were within 1% across all positions. At the isocenter, MAEs increased at 390 mm off‐center positions, with chest showing lateral/vertical errors and abdomen exhibiting longitudinal/rotational deviations. When the beam center was 8 cm lateral and adjacent to a distorted region, errors increased, while contralateral placement showed errors comparable to the isocenter. Maximum RMSEs of 10.98 mm (translation) and 5.88° (rotation) occurred in the abdomen when beam centers overlapped distorted regions, exceeding typical clinical thresholds (5 mm, 2°). The pelvis showed relatively stable alignment with RMSEs within 2.5 mm and 1.5°.

**Conclusion:**

This phantom study demonstrated that eFOV reconstruction can compromise SGRT alignment accuracy, particularly when beam centers are located near distorted regions. Alignment remained within clinically acceptable limits when reference points were sufficiently distant from distortion. These findings provide important baseline data under controlled conditions, and clinical validation with patient cohorts is warranted to confirm their generalizability.

## 1 INTRODUCTION

Recent advances in optical surface imaging (OSI) systems have contributed to the widespread clinical use of surface‐guided radiation therapy (SGRT) across multiple anatomical treatment sites.^1^ The SGRT system enables accurate patient setup using three‐dimensional surface imaging, eliminating the need for permanent skin markings and avoiding additional radiation exposure. It also enables real‐time monitoring of patient positioning during treatment, allowing for quantitative assessment of setup accuracy. The system incorporates an automatic safety interlock function that temporarily interrupts treatment when patient motion exceeds a predefined threshold. These technical capabilities address limitations of conventional tattoo‐based setup methods, such as the risk of skin infections and allergic reactions. They also mitigate the psychological burden and reduced quality of life associated with permanent skin markings.^2^

The accuracy of SGRT implementation depends largely on the reference surface, which serves as the basis for patient alignment. The reference surfaces used in SGRT can be obtained by two methods. The first is based on external body contour generated from computed tomography (CT) data within the treatment planning system. The second involves directly acquiring the patient's surface in the treatment room using an OSI system.^3^ The CT‐based reference surfaces contribute to improved setup accuracy by facilitating reproducible patient positioning consistent with the simulation setup.^4^ Given these advantages, this method is currently the most widely used in clinical practice.^5^ According to a 2022 international survey, 56%–71% of SGRT‐implementing institutions reported using CT‐based reference surfaces across various anatomical regions, including the breast, thorax, and abdomen. In contrast, only 6%–20% of institutions used surfaces acquired at the first or each treatment fraction.

Although CT‐based reference surfaces are widely utilized in clinical practice, their geometric fidelity could be affected by errors introduced during image acquisition. Various imaging parameters and physical factors (respiratory motion, slice thickness, metallic implants, and beam center location) may influence the geometric accuracy of the reconstructed CT images.^6^, ^7^ Such factors can potentially result in artifacts that compromise the accuracy of the external body contour. Since the reference surface in SGRT is commonly generated from this external body contour, such distortions may compromise its geometric integrity. For instance, Meyer et al. demonstrated that SGRT alignment errors could increase by up to 16 mm and 7°, depending on the beam center location. Additionally, Ayrancioglu et al. reported that increased slice thickness was associated with decreased alignment accuracy and recommended using a slice thickness of 3 mm or less.^8^

In addition to acquisition‐related factors, CT reconstruction algorithms may also introduce geometric inaccuracies. One such example is the extended field‐of‐view (eFOV) mode, which is used to reconstruct anatomical structures extending beyond the standard field‐of‐view (sFOV). This mode is often applied in patients with larger body sizes or when immobilization devices are in place. Depending on the manufacturer, this mode may be referred to as high‐definition field‐of‐view (HD FOV) or MaxFOV2. eFOV reconstruction extrapolates truncated projection data beyond the sFOV using a projection‐mass consistency algorithm.^9^ This process estimates the missing information and approximates the patient's external body contour outside the scanned area. This enables the reconstruction of peripheral anatomical regions in the CT images. However, since these regions are generated from estimated rather than measured data, geometric distortions may occur in the reconstructed peripheral anatomical structures.

The geometric inaccuracy of anatomical structures reconstructed in the eFOV region may influence radiotherapy accuracy. However, most previous studies have primarily focused on changes in Hounsfield units (HU) or dose distribution in the eFOV region. Limited attention has been given to the potential impact of geometric distortion on patient alignment accuracy. Beeksma et al. reported that CT numbers varied by up to ± 130 HU depending on the anatomical region of an anthropomorphic phantom.^10^ They also noted that geometric volumes could be overestimated by as much as 1.49%. Furthermore, Wu et al. observed an average volumetric increase of 2.5%–7.5% in the eFOV region, with corresponding dose differences limited to within 3%.^11^ Although geometric distortion associated with eFOV reconstruction may affect SGRT alignment, its quantitative impact and the influence of distortion relative to treatment beam center location have not been comprehensively evaluated.

The purpose of this study was to quantitatively evaluate the impact of CT image distortion caused by eFOV reconstruction on the alignment accuracy of SGRT reference surfaces. To this end, CT scans were acquired under various positional conditions across three anatomical regions. These reference surfaces were subsequently used to compare alignment results between SGRT and cone‐beam CT (CBCT). Furthermore, to evaluate the influence of geometric distortion in the eFOV region, alignment accuracy was assessed with respect to beam center location.

## 2 METHODS

### 2.1 SGRT system

The SGRT system used in this study was Catalyst HD (C‐RAD AB, Uppsala, Sweden), which consists of three scanner units arranged 120° apart around the isocenter.^12^ The system projects a structured light pattern with a wavelength of 405 nm onto the patient's surface and reconstructs a three‐dimensional surface in real time using triangulation of the reflected light. The Catalyst HD system performs surface registration using a non‐rigid registration algorithm. This approach is designed to minimize alignment errors with the reference surface, even in the presence of anatomical deformations.^7^ The camera parameters of the Catalyst HD system were configured with an exposure time of 4000 µs, a gain of 400%, and a surface averaging time of 3 s. The system was thermally stabilized and underwent daily quality assurance prior to each experimental session.

### 2.2 Anthropomorphic phantom setup

**FIGURE 1:**
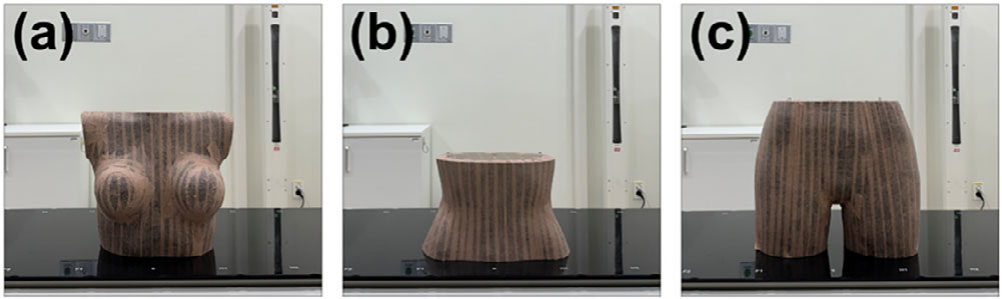
Anthropomorphic phantom segmented into (a) chest, (b) abdomen, and (c) pelvis. Three regions were taped to enhance surface visibility for the SGRT system.

Figure 1 shows an anthropomorphic phantom segmented into three anatomical regions (chest, abdomen, and pelvis). These regions were used to evaluate region‐specific differences in SGRT alignment accuracy. To ensure consistent surface detection by the SGRT system, skin‐colored masking tape was applied to the entire surface of the phantom. The chest and pelvis were immobilized using a vac‐lok cushion to ensure positional stability during setup. A radiopaque marker was attached to the geometric center of each anatomical region and used as a reference point for both CT simulation and patient setup in the treatment room.

### 2.3 CT image acquisition and generation of SGRT reference surface

**FIGURE 2:**
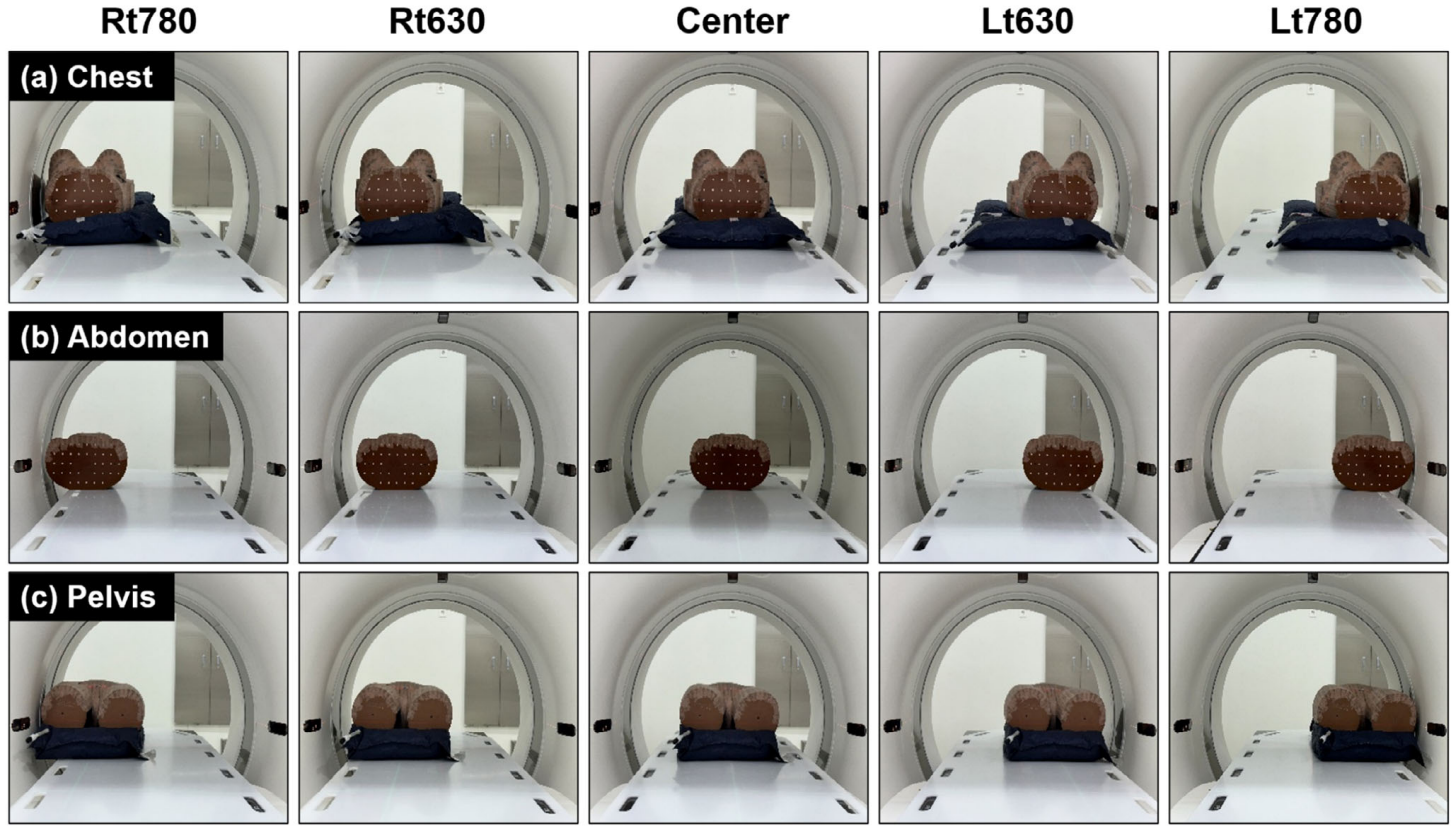
CT setup positions of the anthropomorphic phantom for three anatomical regions at five positions (center and four off‐center). The phantom was scanned in the (a) chest, (b) abdomen, and (c) pelvis configurations at five positions: Rt780, Rt630, Center, Lt630, and Lt780, corresponding to increasing lateral displacement from the CT isocenter.

Figure 2 shows the five CT bore positions evaluated for all three anatomical regions (chest, abdomen, and pelvis). These positions consisted of a Center, where the phantom's central axis was aligned with the CT isocenter, and four off‐center locations (Rt630, Rt780, Lt630, and Lt780). In the off‐center conditions, the outer edge of the phantom was positioned either 315 mm or 390 mm to the left or right of the isocenter, corresponding to effective bore diameters of 630 mm and 780 mm, respectively. These positions were selected to evaluate the effects of image distortion in peripheral and mid‐peripheral regions, where geometric inaccuracies are more pronounced.^11^ CT images were acquired using a Somatom Confidence RT Pro scanner (Siemens Healthineers, Germany). For each position, the phantom was aligned such that the attached radiopaque marker matched the CT simulator's laser isocenter. The CT table was laterally extended using a patient transfer board. Scans were acquired using a consistent protocol: 120 kVp, 400 effective mAs, 2 mm slice thickness, and a pitch of 1.0. The regions of interest (ROIs) were defined as 3 cm shorter than the superior and inferior boundaries of each anatomical region, replicating typical clinical scan conditions. This setup was intended to replicate clinical conditions in which only the target anatomical region is included in the scan range, as typically performed in patient imaging. In the Center, the phantom was fully enclosed within the sFOV, so eFOV reconstruction was not applied. For the off‐center positions, images were reconstructed using the eFOV mode. The phantom was set up once for each position, and five repeated acquisitions were performed without repositioning to ensure consistency in distortion characteristics.

**FIGURE 3:**
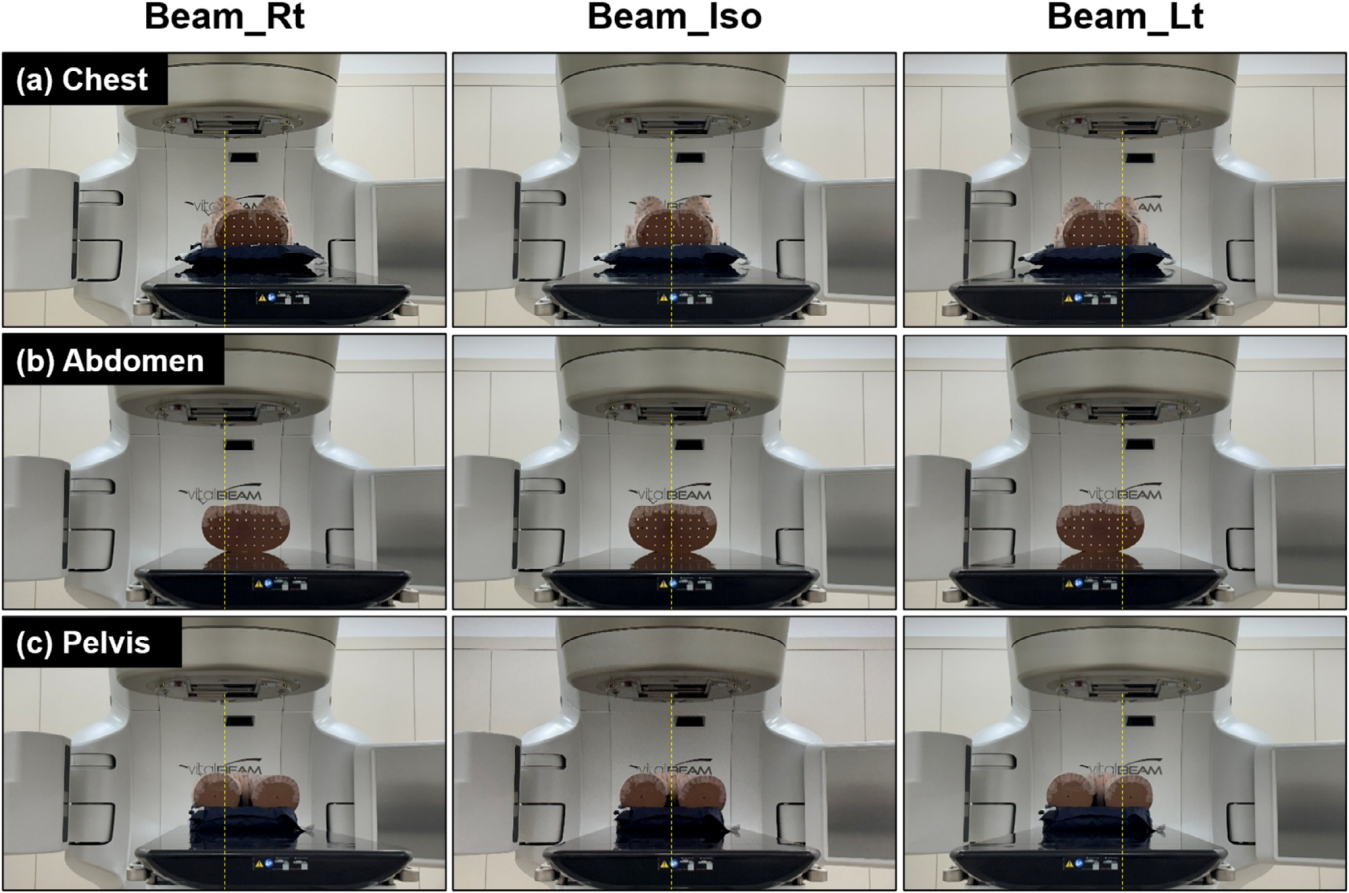
Setup positions at three beam center locations for each anatomical region. Three beam center locations (Beam_Rt, Beam_Iso, and Beam_Lt) were evaluated in (a) chest, (b) abdomen, and (c) pelvis configurations. Phantom alignment was performed using radiopaque markers and in‐room laser systems.

Figure 3 shows the three beam center conditions (Beam_Rt, Beam_Iso, and Beam_Lt) used to evaluate the effect of beam center shifts on SGRT alignment accuracy. Treatment plans were generated under these conditions for each anatomical region. Beam_Iso corresponded to the central position where the radiopaque marker aligned with the treatment room lasers, whereas Beam_Rt and Beam_Lt corresponded to positions shifted 8 cm laterally to the right and left of the isocenter, respectively. The reference surface for the Catalyst HD system was generated from the external body contour automatically created using the body searching tool in the Eclipse TPS (version 16.1; Varian Medical Systems, Palo Alto, CA). The external body contour was generated by applying a threshold of –350 HU to the CT images. To enable consistent volume evaluation across positional conditions, the same longitudinal range was applied when generating the external body contour.

### 2.4 Evaluation of volume distortion

Volume distortion was quantitatively evaluated based on the external body contour generated in the treatment planning system (TPS). For each anatomical and phantom position, the mean and standard deviation (mean  ±  SD) of the contour volume were calculated. The volume measured at the Center was used as a reference, and volume changes at each off‐center position were compared accordingly.

### 2.5 Assessment of SGRT and CBCT alignment errors

The phantom was first positioned at Beam_Iso by aligning the radiopaque marker with the treatment room lasers. Alignment errors were then measured under this setup using both SGRT and CBCT without re‐setup, allowing for evaluation of the reproducibility of each alignment system. The same procedure was repeated at the Beam_Rt and Beam_Lt positions by moving the treatment couch according to the treatment plan. SGRT alignment error (SAE) was calculated as the positional difference between the real‐time surface image and the SGRT reference surface. CT alignment error (CAE) was determined by automatically matching the simulation CT and the acquired CBCT using the entire imaged region as the ROIs.

### 2.6 Analysis of SGRT and CBCT alignment errors

The alignment differences between SAE and CAE were calculated for six directions: three translational (lateral [Lat], longitudinal [Lng], and vertical [Vrt]) and three rotational (yaw, roll, and pitch). These differences were quantified using mean absolute error (MAE) and standard deviation (SD), as defined by the following equations:

(1) $$MAE=\frac{1}{N}\;\sum_{i=1}^{N}\left|SAE_{i,x}-CAE_{i,x}\right|$$

(2) $$\mathrm{SD}=\sqrt{\frac{1}{N-1}\sum_{i=1}^{N}{\left(\mathrm{SA}E_{i,x}-\mathrm{CA}E_{i,x}\right)}^{2}}$$

where *x* denotes the six directions described above, and *N* represents the number of data points for each condition. Translational and rotational root mean square errors (RMSEs) were also calculated using the following equations to evaluate the overall impact of image distortion on SGRT alignment accuracy.

(3) $$Translational\;RMSE=\sqrt{\frac{1}{N}\sum_{i=1}^{N}\left[{\left(SAE_{i,Lat}-CAE_{i,Lat}\right)}^{2}+{\left(SAE_{i,Lng}-CAE_{i,Lng}\right)}^{2}+{\left(SAE_{i,Vrt}-CAE_{i,Vrt}\right)}^{2}\right]}$$

(4) $$Rotational\;RMSE=\sqrt{\frac{1}{N}\sum_{i=1}^{N}\left[{\left(SAE_{i,Yaw}-CAE_{i,Yaw}\right)}^{2}+{\left(SAE_{i,Roll}-CAE_{i,Roll}\right)}^{2}+{\left(SAE_{i,Pitch}-CAE_{i,pitch}\right)}^{2}\right]}$$

where N denotes the number of data points under each condition.

## 3 RESULTS

### 3.1 Evaluation of volume distortion by phantom position

**FIGURE 4:**
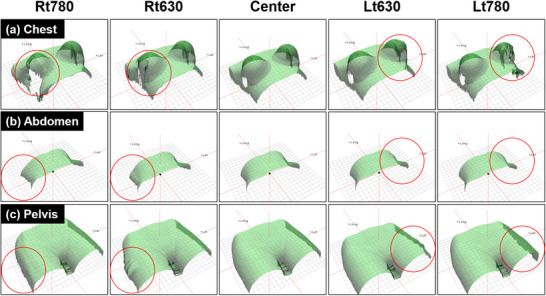
Comparison of external body contours between Center and Lt780 CT scans. Axial and sagittal CT images acquired at the Lt780 position are shown for (a, b) chest, (c, d) abdomen, and (e, f) pelvis. Yellow contours represent external body contours generated from the Center scan, while green contours correspond to those from the Lt780 scan. Off‐center positioning under eFOV conditions led to incomplete reconstruction of the lateral chest wall, localized deformation in the abdomen, and contour underestimation in the pelvis.

Figure 4 shows axial and sagittal CT images of the chest, abdomen, and pelvis acquired at the Lt780. The green and yellow contours represent the automatically generated external body contours from the Lt780 and Center, respectively. In the chest, severe geometric distortion led to incomplete reconstruction of the lateral chest wall, resulting in partial exclusion of the left lung from the external body contour. The abdomen demonstrated localized expansion superiorly and inward deformation inferiorly along the left boundary. In the pelvis, reduced soft‐tissue volume was noted at the iliac crest level and proximal thigh, leading to contour underestimation.

The impact of geometric distortion in each anatomical region was quantitatively evaluated by comparing external body contour volumes generated from the CT images. Table 1 summarizes the mean volume, SD, and percentage volume change relative to the Center for each phantom position and anatomical region. In the chest, the farther the phantom was positioned from the Center, the greater the volume reduction, with a maximum decrease of 12.55% observed at the Rt780. In contrast, volume differences at Rt630 and Lt630 were within 3% of the Center. For the abdomen and pelvis, all positions showed volume changes within ± 1% relative to the Center.

**TABLE 1:** Volume summary at five phantom positions and three anatomical regions.

Region		Center	Lt630	Rt630	Lt780	Rt780
**Chest**	**AV(cc)**	12258.1	11909.5	12042.4	10888.9	10720.0
	**SD(cc)**	45.71	26.21	37.49	59.79	147.57
	**VD(%)**	Reference	−2.84	−1.76	−11.17	−12.55
**Abdomen**	**AV(cc)**	4180.7	4212.4	4217.7	4211.5	4217.9
	**SD(cc)**	9.86	6.88	19.64	16.52	24.83
	**VD(%)**	Reference	0.76	0.89	0.74	0.89
**Pelvis**	**AV(cc)**	13802.8	13871.7	13903.8	13721.9	13739.8
	**SD(cc)**	1.78	1.97	6.91	7.09	9.06
	**VD(%)**	Reference	0.50	0.73	−0.59	−0.46

Average external body contour volume (AV), standard deviation (SD), and volume difference (VD) measured at five phantom positions within the CT bore for the chest, abdomen, and pelvis. VD denotes the percentage difference relative to the Center (reference).

**FIGURE 5:**
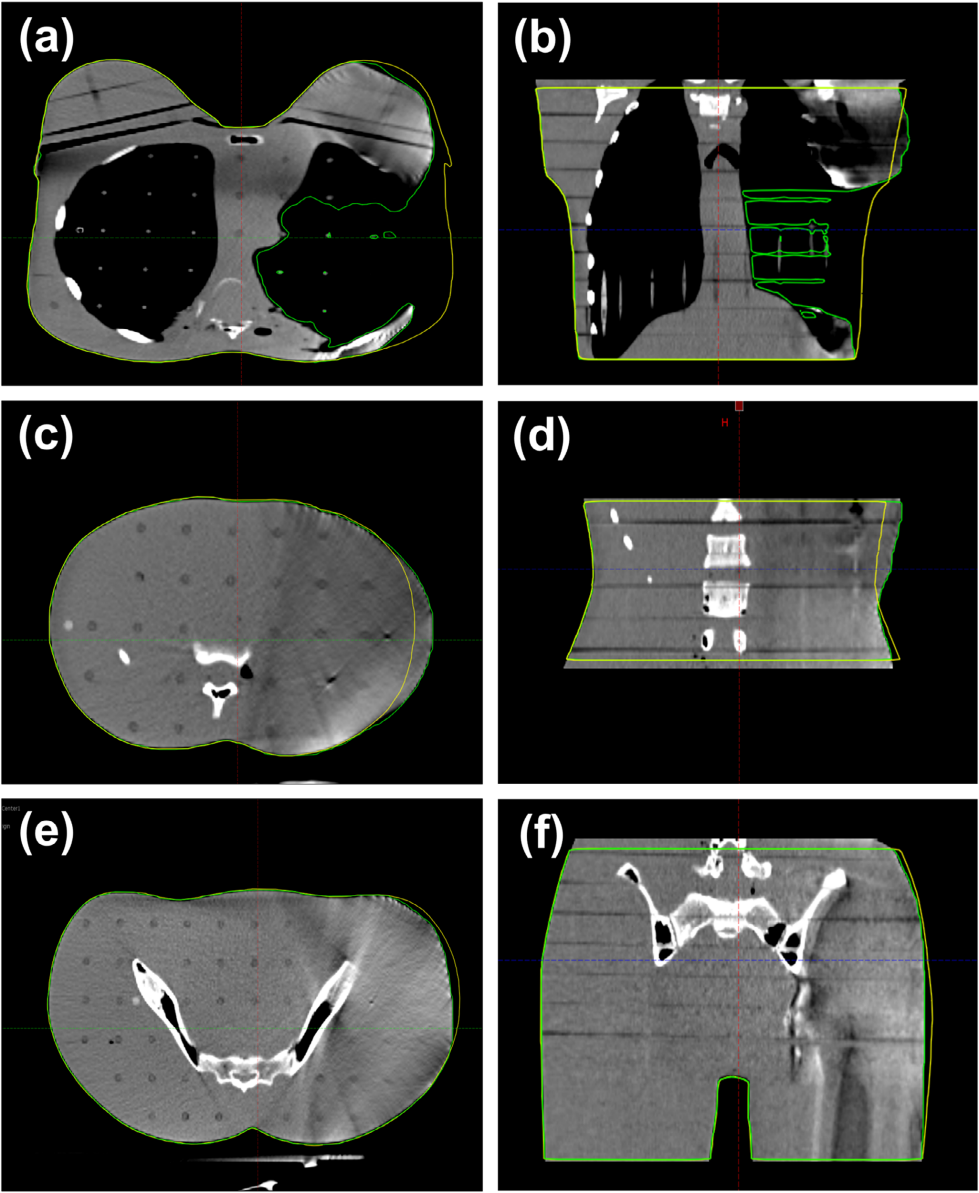
SGRT reference surfaces generated by the Catalyst system for three anatomical regions. (a) chest, (b) abdomen, and (c) pelvis reference surfaces were generated from CT images acquired at five phantom positions: Rt780, Rt630, Center, Lt630, and Lt780. Red circles highlight regions affected by eFOV distortion. These included deformation of the breast and lateral chest wall in the chest, minimal changes in the abdomen, and distortion of the thigh surface in the pelvis.

Geometric distortion observed in CT images was partially reflected in the generation of SGRT reference surfaces. Figure 5 shows the reference surfaces generated by the Catalyst HD system based on CT images. Red circles indicate locations on the reference surface affected by distortion from eFOV reconstruction. In the chest, reference surfaces generated from the Lt780 and Rt780 showed visible deformation. This included distortion of the breast and lateral chest wall, corresponding to the underlying CT distortion. In the abdomen, only minimal shape changes were observed along the left flank within the eFOV region, and these were difficult to distinguish visually. The pelvis showed deformation of the thigh surface, particularly at the Rt630 and Lt630, whereas no apparent distortion was observed at the Rt780 and Lt780.

### 3.2 Directional setup error evaluation using MAE

**FIGURE 6:**
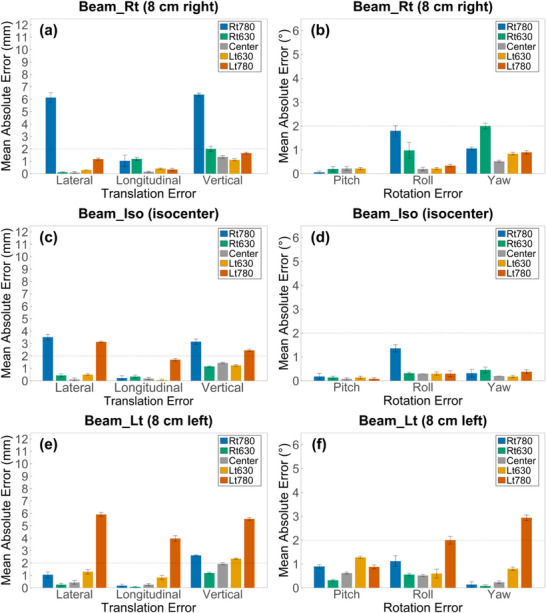
Translational and rotational MAEs in the chest under three beam center locations. (a, c, e) show translational MAEs, and (b, d, f) show rotational MAEs between SAE and CAE under Beam_Rt (isocenter shifted 8 cm to the right), Beam_Iso (isocenter at the center), and Beam_Lt (isocenter shifted 8 cm to the left). Error bars indicate standard deviations.

**FIGURE 7:**
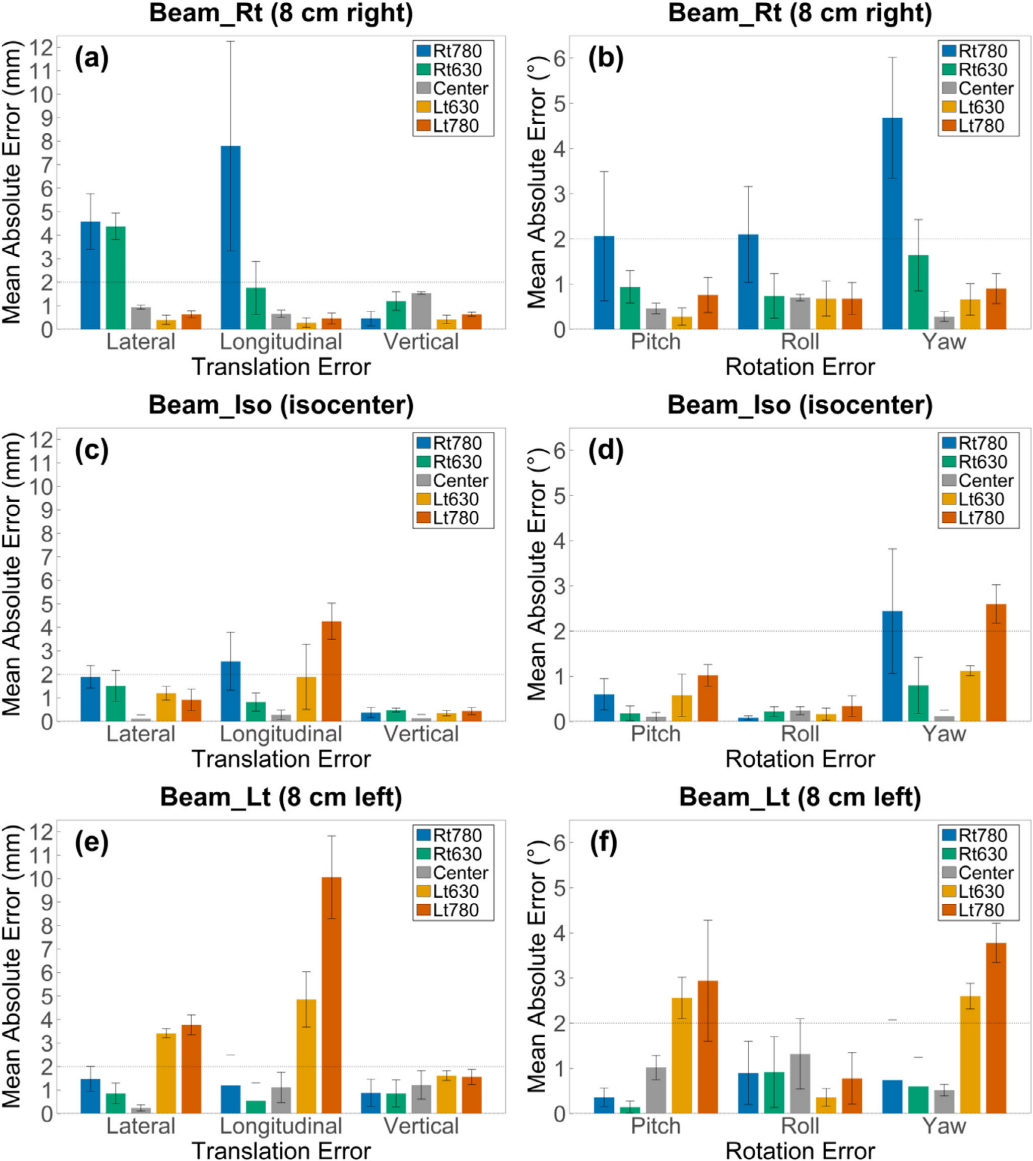
Comparison of translational and rotational MAEs in the abdomen under different beam center locations. (a, c, e) show translational MAEs, and (b, d, f) show rotational MAEs between SAE and CAE under Beam_Rt (isocenter shifted 8 cm to the right), Beam_Iso (isocenter at the center), and Beam_Lt (isocenter shifted 8 cm to the left). Error bars indicate standard deviations.

**FIGURE 8:**
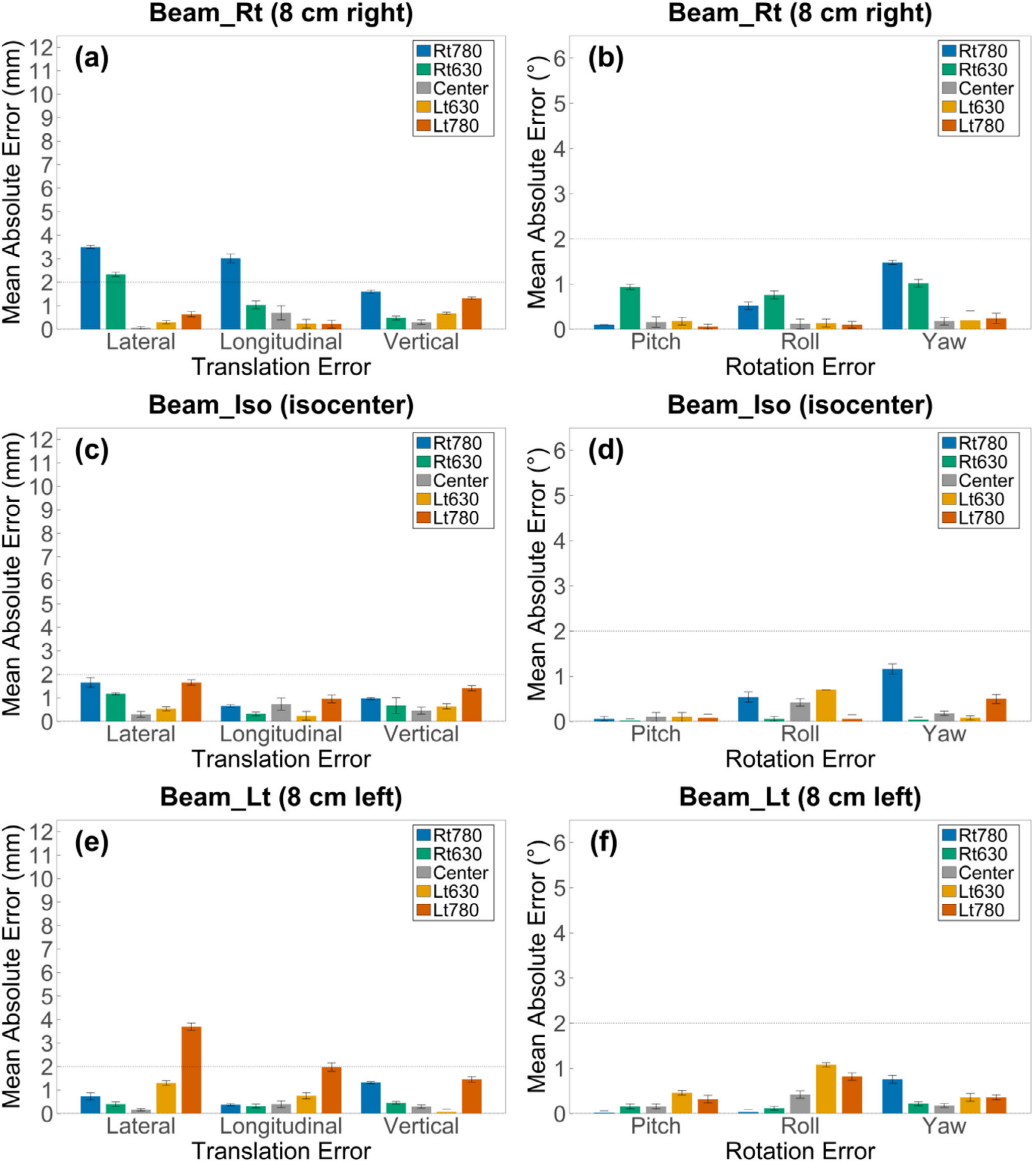
Region‐specific MAEs in the pelvis under three beam center conditions. MAEs between SAE and CAE are shown for (a, c, e) translational and (b, d, f) rotational directions, with standard deviation error bars for each condition. Beam_Rt, Beam_Iso, and Beam_Lt denote the beam isocenter shifted 8 cm to the right, at the center, and shifted 8 cm to the left, respectively.

Figures 6, 7, 8 present MAE and SD for each axis, categorized by anatomical region and beam center condition. All MAE values represent the difference between SAE and CAE.

In the chest, the Center under Beam_Iso showed MAE values within 2 mm and 1° across all axes. However, increased errors were observed in the Lateral and Vertical directions at Rt780 and Lt780. At Rt780 under Beam_Rt, MAEs reached 6.14 mm (Lateral), 6.38 mm (Vertical), and 1.80° (Roll). At Lt780 under Beam_Lt, errors increased across various directions, with MAEs of 5.92 mm (Lateral), 3.98 mm (Longitudinal), 5.56 mm (Vertical), 2.00° (Roll), and 2.94° (Yaw).

In the abdomen, the Center under Beam_Iso maintained MAE values within 2 mm and 2° across all axes. However, MAEs increased in the Lateral, Longitudinal, and Yaw directions at both Rt780 and Lt780. At Rt780 under Beam_Rt, MAEs reached 4.58 mm (Lateral), 7.80 mm (Longitudinal), and 4.68° (Yaw). At Lt780 under Beam_Lt, the corresponding values were 3.78 mm, 10.06 mm, and 3.78°, respectively.

In the pelvis, under Beam_Iso, MAE values were maintained within 2 mm and 2° across all position. At Rt780 under Beam_Rt, MAEs were 3.50 mm (Lateral), 3.02 mm (Longitudinal), and 1.60 mm (Vertical). At Lt780 under Beam_Lt, MAEs were slightly elevated to 3.70 mm (Lateral), 1.98 mm (Longitudinal), and 1.46 mm (Vertical).

### 3.3 Evaluation of overall setup accuracy using RMSE

**FIGURE 9:**
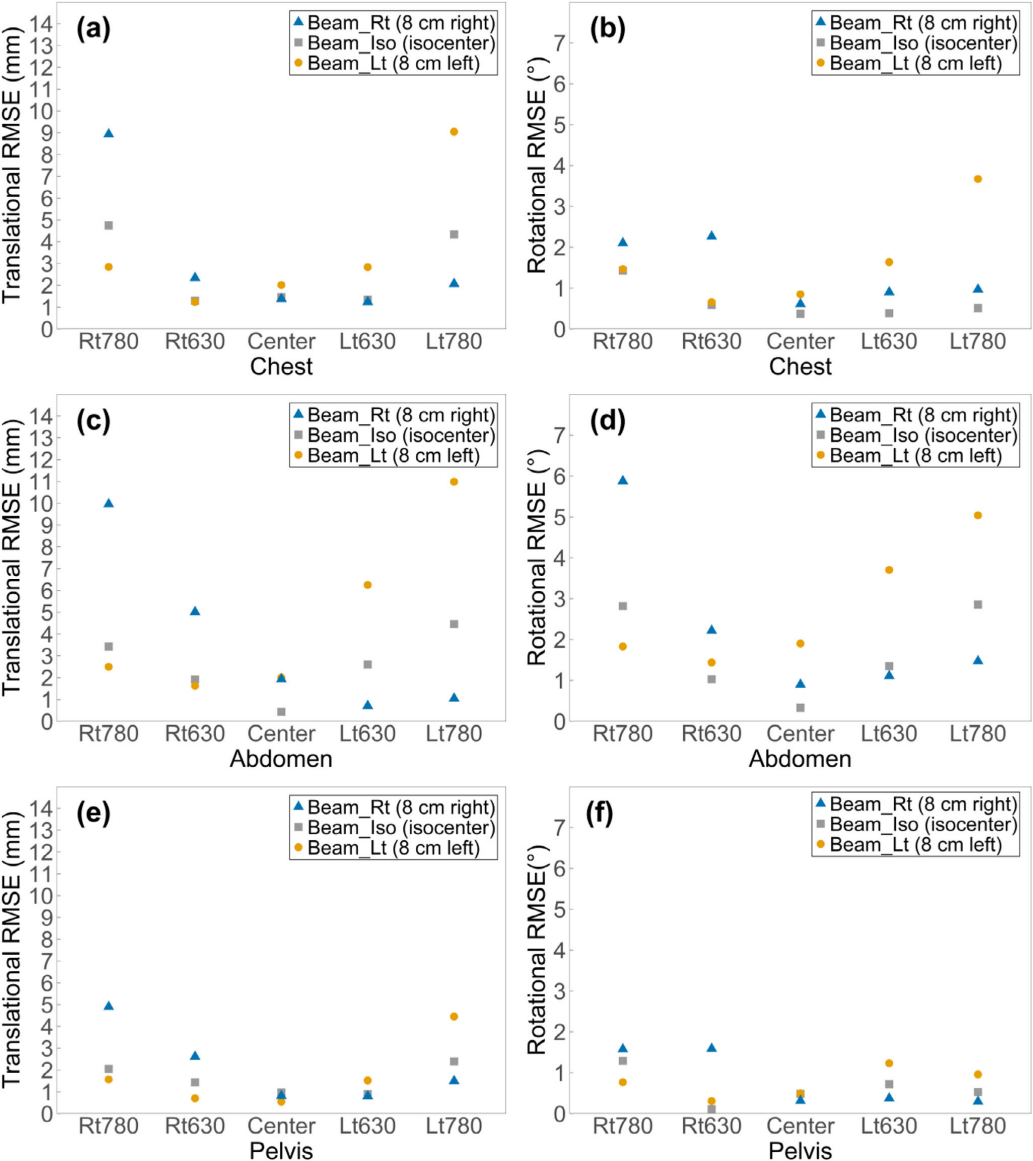
RMSE of SGRT–CBCT setup deviations across three anatomical regions and beam center conditions. RMSE values of translational and rotational setup deviations between SAE and CAE are shown across three anatomical regions (chest, abdomen, and pelvis) and beam center conditions: Beam_Rt (8 cm right), Beam_Iso (isocenter), and Beam_Lt (8 cm left).

Figure 9 presents the translational and rotational RMSE values across various phantom positions and beam center configurations for each anatomical region.

In the chest, at the Center under Beam_Iso, translational and rotational RMSEs were 1.46 mm and 0.37°, respectively, with similarly low values observed at Rt630 and Lt630. In contrast, RMSEs increased at Rt780 and Lt780, with translational values of 4.75 mm and 4.34 mm and rotational RMSEs of 1.43° and 0.51°, respectively. Under Beam_Rt, translational and rotational RMSEs at Rt780 were 8.93 mm, and 2.10°, respectively. In contrast, RMSEs at Lt780 were relatively low. Under Beam_Lt, translational and rotational RMSEs at Lt780 increased to 9.05 mm and 3.67°, respectively. In contrast, RMSEs at Rt780 showed only a slight increase compared to those at the Center.

In the abdomen, RMSEs increased more substantially than in the chest under comparable conditions. At the Center under Beam_Iso, translational and rotational RMSEs were 0.43 mm and 0.33°, respectively. Both values increased progressively as the phantom moved toward the periphery. At Rt780 and Lt780, translational RMSEs increased by factors of 7.95 and 10.33, and rotational RMSEs by 8.50 and 8.61, respectively, compared to the Center. Under Beam_Rt, RMSEs at Rt780 reached 9.96 mm (translation) and 5.88° (rotation), while values at Lt780 remained relatively low. Under Beam_Lt, RMSEs at Lt780 increased to 10.98 mm (translation) and 5.04° (rotation), whereas values at Rt780 were comparable to those at the Center.

In the pelvis, translational and rotational RMSEs were generally smaller than those observed in the chest and abdomen. Under Beam_Iso, RMSEs at the Center were 0.97 mm (translation) and 0.48° (rotation). Slight increases were observed at Rt780 (2.05 mm, 1.29°) and Lt780 (2.39 mm, 0.53°). Under Beam_Rt, RMSEs at Rt780 increased markedly to 4.90 mm (translation) and 1.57° (rotation), while values at Lt780 remained below 2.00 mm and rotational RMSEs were comparable to the Center. Under Beam_Lt, RMSEs at Lt780 increased to 4.45 mm (translation) and 0.96° (rotation), whereas values at Rt780 remained close to those at the Center.

## 4 DISCUSSION

This study quantitatively evaluated the impact of image distortion associated with eFOV reconstruction on SGRT alignment accuracy. To minimize variability due to setup reproducibility, all CT image acquisitions and SGRT alignment procedures were conducted under identical setup conditions without repeated repositioning of the phantom. In addition, five repeated CT scans were acquired at each position to evaluate the consistency of both image distortion and alignment errors.

At the Rt780 in the chest, the volume was reduced by up to 12.55% compared to the Center. This reduction may be associated with geometric distortion in the reconstructed chest wall. Partial exclusion of the outer surface could have led to discontinuity between the lung and surrounding air, potentially causing underestimation of the lung contour. Consequently, the lung region was not included in the external body contour generated from the distorted CT image. According to a vendor white paper on eFOV reconstruction, geometric distortion is more likely to occur when low‐density structures such as the lungs are positioned near the edge of the sFOV (within the 50 cm bore diameter).^13^ This effect is particularly pronounced when these structures are adjacent to high‐density structures beyond the sFOV boundary. Notably, the greatest volume reductions were observed at Rt780 and Lt780 in the chest. In contrast, distortion was less pronounced at the Lt630 and Rt630, which may be attributed to the spatial relationship between the reconstructed FOV boundary and the surrounding anatomical structures. Wu et al. observed volume increases of approximately 7.5% in the chest region.^11^ However, our study demonstrated a reduction in chest volume, likely resulting from incomplete reconstruction of the chest wall and partial exclusion of lung structures. Therefore, in the chest, additional consideration may be warranted regarding the selection of contouring thresholds within the treatment planning system.

Volume changes in the abdomen and pelvis were limited to within 1%. However, as shown in Figure 4, localized expansion and contraction due to image distortion were observed near the outer surface of the phantom. This suggests that even minor overall volume changes may affect the reference surface if geometric distortion is present. Beeksma et al. reported volume overestimation by up to 1.5% within the eFOV region in pelvis phantoms.^10^ This was consistent with our findings at Lt630 and Rt630 in the abdomen and pelvis. However, at Rt780 and Lt780 in the pelvis, we observed volume reductions rather than increases. These discrepancies may be attributed to differences in eFOV reconstruction algorithms and the specific positioning of anatomical structures within the eFOV region. The AAPM TG‐302 report further notes that various factors (motion artifacts, slice thickness, metallic implants, and immobilization devices) could affect the geometric accuracy of CT‐based reference surfaces, potentially reducing the alignment accuracy of SGRT.^6^

Such volume changes may be reflected in the SGRT reference surface and contribute to alignment errors. When distorted CT images acquired with eFOV reconstruction were used as reference surfaces, alignment errors increased. Both translational and rotational MAEs rose under certain conditions across all three anatomical regions. At the Center, alignment errors remained within 2 mm and 2°, regardless of beam center location. In contrast, under Beam_Rt or Beam_Lt conditions, translational MAEs increased up to 10.06 mm and rotational MAEs up to 4.68°. Alignment error patterns varied by anatomical site. The chest showed greater deviations in the lateral and vertical directions, while the abdomen exhibited larger errors in the longitudinal and yaw axes. In contrast, the pelvis demonstrated relatively uniform deviations across all directions. These axis‐dependent trends may reflect anatomical geometry and its interaction with surface distortion during eFOV reconstruction.

A similar trend was observed in the RMSE analysis, which further characterized overall alignment deviations. Under Beam_Iso, RMSEs at the Center were maintained within 1.5 mm and 1° across all three anatomical regions. In contrast, when the phantom was positioned near the 780 mm boundary, translational and rotational RMSEs increased, reaching up to 4.75 mm and 2.85°, respectively. The pelvis exhibited relatively stable alignment accuracy, with RMSEs maintained within 2.5 mm and 1.5° across all positions. These findings suggest that geometric distortion within the eFOV region could affect alignment accuracy differently depending on the anatomical site and phantom position.

Recent studies have established SGRT positioning thresholds of 3 mm and 2° for patient setup, whereas some institutions use more lenient tolerances up to 5 mm and 2°.^14^, ^15^, ^16^ At these higher tolerance levels, translational and rotational RMSEs in the chest and abdomen exceeded 5 mm and 2° under certain eFOV conditions, particularly when the beam center was located near the region of distortion (Rt780 and Lt780). In contrast, the pelvis showed smaller alignment deviations overall, although translational RMSEs approached 5 mm when the beam center was located near the distortion boundary, as in the Rt780 and Lt780. These findings indicate that translational and rotational RMSEs exceeded commonly accepted thresholds under specific eFOV conditions, suggesting that reference surface deformation caused by image distortion may significantly compromise alignment accuracy.

This study represents one of the initial quantitative analyses to investigate the impact of eFOV on SGRT alignment accuracy. It focused on differences in alignment errors according to phantom position. The observed increases in alignment errors suggest that accuracy is influenced not only by the presence of image distortion but also by its location and pattern within the reference surface. In addition, the relative position of the beam center was identified as a critical factor influencing alignment accuracy. These findings are consistent with Meyer et al., who reported SGRT alignment errors of up to 16 mm and 7° when surface deformations were present, depending on beam center location.^7^ In that study, anatomical deformations were evaluated, whereas our study examined eFOV reconstruction artifacts. Both demonstrate that beam center proximity to distorted regions significantly impacts alignment accuracy. Therefore, the characteristics of image distortion should be considered prior to generating the SGRT reference surface. Under certain conditions, supplementary alignment methods, such as tattoo‐laser setup or adjustment of the SGRT ROIs, may be warranted. Importantly, the AAPM TG‐302 report explicitly states that when CT‐based surface images are used in SGRT systems, adequate image quality must be ensured.^6^ This supports the importance of evaluating image distortion before generating the SGRT reference surface.

Moreover, alignment outcomes may vary across SGRT systems, as different vendors implement distinct registration algorithms. Pallotta et al. demonstrated with a deformable phantom that non‐rigid registration achieved better alignment accuracy than rigid registration under realistic deformation conditions.^17^ This suggests that the choice of registration algorithm may influence alignment performance and should be considered when generalizing these results across platforms.

This phantom‐based study provided controlled assessment of eFOV distortions but excluded patient‐specific factors such as respiratory motion, body habitus variations, and inter‐fractional positioning changes. These factors, particularly respiratory motion in thoracic treatments, could introduce additional geometric uncertainties beyond our measured values. Therefore, our findings should be interpreted as baseline measurements under controlled conditions, with clinical implementation requiring consideration of these additional variables.

This study employed measurements of a single phantom across multiple positions and conditions, with repeated acquisitions to ensure reproducibility. Given this single‐phantom design with repeated measurements, descriptive error metrics (MAE and RMSE) were used for direct comparison with established tolerance thresholds. This approach provided baseline data on eFOV‐related distortion patterns for SGRT applications. Future work should include validation of these findings in patient cohorts and confirmation across different SGRT systems and registration algorithms. In addition, multi‐institutional studies with larger sample sizes are needed to enable formal statistical testing and to investigate correlations between distortion patterns and alignment errors.

## 5 CONCLUSION

This study evaluated the impact of CT image distortion from eFOV reconstruction on the alignment accuracy of SGRT reference surfaces. Optimal alignment was consistently achieved when the phantom was positioned at the CT bore center, regardless of beam center location. In contrast, when any part of the phantom extended into the eFOV region, alignment errors increased. We observed the largest errors when the beam center was located within or near distorted regions. Conversely, alignment accuracy remained clinically acceptable when the reference point was sufficiently distant from these regions.

These results underscore potential clinical considerations when generating SGRT reference surfaces using eFOV reconstructions. Our findings suggest that positioning treatment sites near the bore center may mitigate the impact of distortion. While not directly evaluated in this study, supplemental approaches such as tattoo‐based setup or CBCT verification may further enhance reliability. As these findings are based on controlled phantom measurements, clinical validation with patient cohorts is warranted to establish optimal protocols for SGRT implementation under eFOV reconstruction.

## AUTHOR CONTRIBUTION

Wooseok Kim contributed to data acquisition, data analysis, and initial drafting of the manuscript. Hyunsoo Jang contributed to study conception, clinical guidance, and critical manuscript revision. Eng Chan Kim contributed to methodology development and technical analysis. Jonggeun Baek contributed to study design, data interpretation, and manuscript revision, and served as a corresponding author. Byungyong Kim contributed to study conception, supervision, and final approval of the manuscript, and served as a corresponding author.

## CONFLICT OF INTEREST STATEMENT

The authors declare that there are no conflicts of interest relevant to this work.
